# Is there a place for a molecular diagnostic test for pelvic inflammatory disease in primary care? An exploratory qualitative study

**DOI:** 10.1371/journal.pone.0274666

**Published:** 2022-09-19

**Authors:** Helen Bittleston, Jane S. Hocking, Jane L. Goller, Jacqueline Coombe, Deborah Bateson, Sally Sweeney, Kirsteen Fleming, Wilhelmina M. Huston

**Affiliations:** 1 Melbourne School of Population and Global Health, The University of Melbourne, Melbourne, Victoria, Australia; 2 Family Planning NSW, Newington, New South Wales, Australia; 3 Specialty of Obstetrics, Gynaecology and Neonatology, Faculty of Medicine and Health, The University of Sydney, Sydney, New South Wales, Australia; 4 Faculty of Science, School of Life Sciences, University of Technology Sydney, Sydney, New South Wales, Australia; St George’s University of London, UNITED KINGDOM

## Abstract

**Introduction:**

There is currently no test for pelvic inflammatory disease (PID) that is non-invasive and sufficiently sensitive and specific. Clinicians must therefore diagnose PID clinically, ruling out medical emergencies and conducting pelvic examinations where possible. While guidelines state that clinicians should be prepared to over-diagnose PID, it remains an under-diagnosed condition, with severe reproductive health impacts when left untreated. This research is the first to consider the perspectives of end-users on the development of a diagnostic test for PID.

**Methods:**

Semi-structured live video feed online (Zoom) interviews were conducted with 11 clinicians and nine women (aged 18–30 years) in Australia to understand how a diagnostic test might be used, and what characteristics a test would need for it to be acceptable to clinicians and young women. Participants were recruited via researcher and university student networks. Reflexive thematic analysis was used to identify key themes relating to the acceptability and characteristics of a diagnostic test for PID.

**Results:**

Seven general practitioners, four clinicians working in sexual health clinics, and nine young women (aged 21–27 years) were interviewed. Clinicians were aged between 31–58 years and were predominantly female. Clinicians recognised that the development of an accurate test to diagnose PID would be valuable to themselves and other clinicians, particularly those who lack experience diagnosing PID, and those working in certain settings, including emergency departments. They discussed how they might use a test to enhance their clinical assessment but highlighted that it would not replace clinical judgement. Clinicians also considered how a test would impact the patient experience and time to treatment, emphasising that it should be minimally invasive and have a quick turnaround time. Young women said a test would be acceptable if endorsed by a trustworthy clinician.

**Conclusions:**

PID remains a challenging diagnosis. Development of a minimally invasive and sufficiently accurate diagnostic test would be acceptable to young women and benefit some clinicians, although no test would completely replace an experienced clinician’s judgement in making a PID diagnosis.

## Introduction

Pelvic inflammatory disease (PID) is a syndrome comprising a spectrum of inflammatory disorders of the upper genital tract, including endometritis, salpingitis, tubo-ovarian abscess and pelvic peritonitis [1]. As PID is not a notifiable disease in Australia, incidence data in general practice and other primary care settings is scare. However, an analysis of hospital admissions data from 2009–2014 has identified an increase in PID diagnoses in the emergency department (ED) [2]. PID can have severe reproductive consequences if not detected and treated, including infertility, ectopic pregnancy, and chronic pelvic pain [3].

PID is an underdiagnosed condition, in part due to the challenges associated with diagnosis, namely the wide variety of clinical presentations and reliance on a subjective clinical diagnosis [4]. There is currently no diagnostic test that is both non-invasive and sufficiently sensitive and specific for PID [1]. While laparoscopy can be used to diagnose PID, it is neither feasible nor justifiable in the vast majority of cases, being an invasive, often costly procedure that may not detect lower levels of fallopian tube inflammation or endometritis [1, 3, 4]. An ultrasound (preferably transvaginal) may be conducted to identify tubo-ovarian abscesses or other serious features of PID, or to exclude other differential diagnoses [1, 5]. However, this should not delay timely treatment, as delays can negatively impact fertility [6].

Clinicians should consider PID in all young women presenting with new onset pelvic pain [1]. After excluding medical emergencies (e.g. ectopic pregnancy, appendicitis), guidelines recommend a speculum examination to visualise the cervix and collect swabs for sexually transmissible infections (STIs), and a bimanual pelvic examination to detect cervical motion tenderness, and/or uterine or adnexal tenderness [5]. Although the presence of an STI is supportive of a PID diagnosis, in most cases no organism is identified, and so the absence of an STI should not prevent a diagnosis [5]. Where a pelvic examination is not possible, a presumptive diagnosis based on history is acceptable. As PID can have serious reproductive health impacts including infertility, guidelines support clinicians to have a low threshold for diagnosis [4, 5].

There is considerable variability between clinicians with regards to the diagnostic threshold for PID. In a study conducted at an Australian sexual health clinic, clinicians diagnosed PID at different rates, suggesting some of the clinicians may have under-diagnosed PID [7]. Furthermore, findings from UK-based research suggest that variability may be related to clinician experience; inexperienced clinicians were found to have lower levels of diagnostic acumen, both under and over-diagnosing PID [8]. Our recent research also suggests that some Australian general practitioners (GPs) lack experience and confidence with interpreting findings from bimanual pelvic examinations, an important component of the clinical diagnosis [9].

The development of an accurate and minimally invasive diagnostic test for PID has been identified as a clinical and research priority [10]. By exploring the perspectives of two groups of potential end-users of a test (young women [patients] and clinicians), this research seeks to understand whether a diagnostic test for PID would be useful and acceptable to end-users, and how clinicians would use this type of test in their practice. While sub-clinical or ‘silent’ PID can occur, [11] this paper focuses on clinician and patient perspectives on the use of a test for symptomatic patients.

## Materials and methods

This study involved interviews conducted online via Zoom (Zoom Video Communications Inc.) throughout October to November 2020 with clinicians and young women in Australia. Interviews with both participant groups were undertaken concurrently. Ethics approval was granted by the University of Technology Sydney Human Research Ethics Committee (Approval number: ETH20-5231). As interviews were not conducted face-to-face, verbal rather than written consent was obtained from all participants before commencement of the interview. A verbal consent script was read out loud to participants, and their consent was audio-recorded. This procedure of obtaining consent, including the verbal consent script, was approved by the ethics committee.

Interview schedules for both clinicians and young women were developed with input from all authors, including clinician investigators. All interviewees were informed that a test for PID was in development, and the method of specimen collection would likely be a clinician-collected cervical or high vaginal swab. Interviewees were told that the possibility for a patient-collected swab would also be investigated.

We conducted 11 semi-structured interviews with clinicians to understand whether they might use a diagnostic test for PID, and to identify key characteristics a test would need to be acceptable and useful. Clinicians were asked when they would consider offering a test, how a test might change their patient management, and what characteristics (e.g. cost to patient, turnaround time) they would require from a test. Clinicians currently working in Australian general practice, family planning and/or sexual health clinics (settings where PID is commonly diagnosed) were eligible to participate [12]. As authors had existing networks within Australian primary care, we decided upon a convenience sampling strategy to minimise costs and expediate data collection. This was achieved through the circulation of a recruitment flyer and email invitation among the researchers’ professional networks.

Semi-structured interviews were also conducted with nine young women to explore the acceptability of a diagnostic test for PID from the patient perspective. Sexually active women living in Australia, aged between 18–30 years (the age group at high risk of PID) were eligible. Interested women who emailed the researchers were asked to confirm that they fit the eligibility criteria prior to commencing an interview. A convenience sample was also utilised for this participant group due to our high level of accessibility to potential participants via university networks, and young women were recruited via University of Technology Sydney (UTS) student noticeboards and email lists. Women interviewed were not required to have been diagnosed with or have any prior knowledge of PID, and were not asked to disclose any personal or medical history. Women were provided with an overview of PID, including causes, symptoms, and health outcomes. They were given the opportunity to ask questions throughout the interview, and the interviewer frequently checked with participants to see if they had understood information given to them, and asked whether they had any questions. Participants were then presented with several hypothetical scenarios (e.g. visiting their GP after experiencing symptoms of PID, being diagnosed with PID, being offered a pelvic examination and test for PID) and were asked to comment on how they might feel and respond in these situations. An overview of the interview guides is available in S1 File.

Recruitment flyers directed interested individuals to contact the researchers, who provided potential participants with a participant information sheet. Those who agreed to participate organised a time with the researchers for an interview conducted via Zoom that they could join online (video optional) or via telephone. Young women were reimbursed for their time with a $30 gift voucher, and clinicians with a $100 gift voucher, in recognition of their time and professional expertise that they would be drawing on for this study. Recruitment continued within both groups of study participants until the research team felt that sufficient data had been collected to answer the research questions.

Audio recordings of interviews were transcribed verbatim and uploaded into NVivo 12 qualitative analysis software [13]. All interviews were analysed by one of the authors (HB) using reflexive thematic analysis, [14, 15] with an inductive approach, allowing the content of the data to direct the development of coding and themes. Analysis began with a process of familiarisation with the data, involving the reading through of transcripts and listening to audio recordings. Initial codes were developed and linked, resulting in the development of themes. These themes were discussed with four of the co-researchers (JC, JLG, JSH, WMH) resulting in further refinement and development of theme names. Themes relating to the acceptability and useability of a test when managing a symptomatic patient are presented here. Illustrative quotes are included in tables, some quotes have been truncated for clarity.

## Results

The demographics of the clinicians and young women interviewed are presented in Table 1. Most clinician participants were female, worked in general practice and over half diagnosed PID frequently (at least once a month). All participants from the ‘young women’ participant group were students living and studying in metropolitan New South Wales. Most were aged between 18–24 years, and most were born in Australia. Of the three who were born overseas, two were born in Asia, and one in Europe.

**Table 1:** Characteristics of interviewees.

**Clinicians**		**n**	**%**
		**(N = 11)**	
**Gender**	Female	10	90.9
	Male	1	9.1
**Age (years)**	30–39	4	36.4
	40–49	3	27.3
	50–59	4	36.4
**State**	Australian Capital Territory	1	9.1
	New South Wales	4	36.4
	Northern Territory^a^	1	9.1
	Queensland	2	18.2
	Tasmania	1	9.1
	Victoria	1	9.1
	Western Australia	1	9.1
**Role**	General practitioner^b^	7	63.6
	Sexual health physician	2	18.2
	Nurse practitioner (sexual health clinic)	2	18.2
**Frequency diagnoses PID**	At least once a month	6	54.5
	Less than once a month, but more than one a year	3	27.3
	Once a year or less	2	18.2
**Young women**		**n**	**%**
		**N = 9**	
**Age (years)**	18–24	7	77.8
	25–30	2	22.2
**Country of birth**	Australia	6	66.7
	Another country	3	33.3
**Employment**	Unemployed	2	22.2
	Part time/casual work	7	77.8
**Course enrolled in**	Undergraduate degree	7	77.8
	Postgraduate degree	2	22.2
**State**	New South Wales	9	100.0

^a^Clinician recently started working in Queensland, but primarily spoke about experience working in Northern Territory

^b^One general practitioner also worked part time in a sexual health clinic

### Interviews with clinicians

Four themes were identified and outlined below from interviews with clinicians. See Table 2 for illustrative quotes.

**Table 2:** Themes and illustrative quotes from interviews with clinicians.

Theme	Illustrative quotes
**There is room to improve the diagnosis of PID**	*I think anything that helps you in the diagnosis of PID is going to be good*, *really*. *Because we’ve*, *sort of*, *learnt to deal with it*, *but it’s not ideal*. *I mean*, *we’ve learnt to manage it in our own way*. *But we don’t have good tools to do that*. #1 (Female/50-59/Sexual Health Clinic/VIC)
	*It would be great to have another tool* […] *because it always feels like an uncertain diagnosis* #2 (Female/30-39/General Practice/TAS)
	*I’ve been in this field forever and I guess I am pretty confident in my diagnosis of PID*, *but we see all these horror stories coming from other places where things are missed and tests aren’t done properly* […] *and one of my real issues is*, *we have a very high rate of appendicectomies in young girls that present to our emergency departments with abdominal pain*. *Nobody even asks them if they’re having sex*, *they just rip their appendix out and then they find* […] *all this peritoneal fluid and sometimes send it off and find that the person’s got PID but they’ve had their appendix ripped out for no good reason*. *Just without assessment*, *and that happens a lot*, *I can tell you*. #11 (Female/50-59/Sexual Health Clinic/WA)
	*I think it would certainly be of value in places like remote area health*, *where PID is a diagnosis that’s often missed in remote areas*. *I know there’s been studies done on that before*. #10 (Female/40-49/Sexual Health Clinic/ACT)
	*Out in rural Australia or something*, *where you can’t get people who’ve got a lot of experience to work*. *You know*, *you could maybe have a more junior person who doesn’t have a great deal of experience* […] *and nurse practitioners and other people who don’t necessarily have a lot of experience with PID* […] *it would be particularly good in those situations*. #1 (Female/50-59/Sexual Health Clinic/VIC)
**A test could assist clinicians with diagnosis, but wouldn’t replace clinical judgement**	*I think I would be more comfortable about my diagnosis* #2 (Female/30-39/General Practice/TAS)
	*If they had the classic symptoms of PID*, *it would probably be useful* […] *just to be able to confirm your diagnosis*. #4 (Female/40-49/General Practice/QLD & NT)
	*I think*, *to be honest*, *I’d probably continue the treatment if I had really clinically thought they had it*, *despite a negative test*. *That would depend on how good your test was*, *I guess*. #7 (Female/50-59/General Practice/NSW)
	*I still don’t think that if you had a clinically severe PID*, *that you would let a negative test stop you from treating*. *But*, *yeah*, *that positive test in a borderline patient would be really useful*. #8 (Female/30-39/General Practice & Sexual Health Clinic/QLD)
**Impact of a test on time to treatment**	*The other really important part of it would be the*, *how quick the turnaround is I guess*, *because if the test*, *you know*, *take a few days then it will be*, *I guess I will say not useful at all*. *Because usually we’ll treat them immediately*. *So it would have to be a test that’s quite quick*. #6 (Male/30-39/Sexual Health Clinic/NSW)
	*If it’s definitely that* [a point of care test], *then you save the cost and inconvenience of going for an ultrasound or any delay*, *that would be a game changer*. *Absolutely*. *We love that stuff*. *As would every emergency department in Australia*, *and around the world*. #7 (Female/50-59/General Practice/NSW)
	*If you’re not suspecting acute infective PID*, *then you can go ahead and treat their chronic pain more effectively a lot earlier*. *Because some of these girls do get bounced from acute department to acute department*, *never getting to the actual real underlying cause because we’re all treating defensively*. #3 (Female/50-59/General Practice/NSW)
**The patient experience is an important consideration for clinicians**	*If it* [the swab] *had some particular*, *you know*, *pipe cleaner wire structure to it that was particularly unpleasant*, *I’d think I’d want to know about that*. #7 (Female/50-59/General Practice/NSW)
	*It feels very contradictory in front of a patient as well when you’ve really decided strongly to do something and then a test will contradict you*, *and then you’re trying to explain*, *“yeah*, *but the test isn’t 100%* [accurate]*”*, *and they’re thinking well*, *“why did you do that anyway then*? *What’s the point*?*”* #3 (Female/50-59/General Practice/NSW)
	*My patients in the sexual health clinic wouldn’t be able to pay anything majority of the time*, *because we’re looking at a very different demographic than I have in my private clinic which is mostly private-billing patients*. *Usually*, *for most of my patients*, *a test $50 and under*, *I could easily convince without much of an issue*. #8 (Female/30-39/General Practice & Sexual Health Clinic/QLD)

Quotes accompanied by clinician number, gender, age group (years), place of work, State/Territory (ACT = Australian Capital Territory, NSW = New South Wales, NT = Northern Territory, TAS = Tasmania, QLD = Queensland, VIC = Victoria, WA = Western Australia)

#### There is room to improve the diagnosis of PID

Clinicians had varying levels of confidence diagnosing PID. Some, particularly those who diagnosed PID frequently (e.g. fortnightly, or monthly), said that they felt reasonably confident in their ability to diagnose PID. Others were less confident, stating that they often question a PID diagnosis. It was widely accepted by clinicians with all levels of experience that a clinical diagnosis is challenging for many, particularly for those who diagnosed PID less often, and ongoing work to develop a test that can provide a definitive result was welcomed. It was agreed that a highly accurate, affordable (preferably government-subsidised) test that is minimally invasive would be beneficial.

While clinicians generally thought a test to improve the diagnosis of PID would be valuable in their own practice, most identified other settings where PID is under-diagnosed where a test could be particularly impactful. Hospital emergency departments (EDs) were most commonly mentioned, with several clinicians discussing how challenging it is for ED clinicians to manage young women presenting with lower abdominal pain. Several interviewees were also aware that PID is underdiagnosed in remote and rural Australia, and suggested clinicians working in these areas may find a diagnostic test useful. Other clinical settings with high proportions of patients at increased risk of STIs and PID, including after-hours and outreach clinics, were also identified as settings where a diagnostic test might be especially helpful.

#### A test could assist clinicians with diagnosis, but wouldn’t replace clinical judgement

Clinicians considered how a test might assist them with diagnosis and were clear that accuracy was important, and a test would only be used if they considered it to be sufficiently discriminatory. Clinicians were asked about desired levels of sensitivity and specificity. Some were hesitant to provide levels of sensitivity and specificity that would be acceptable to them, but those who did generally said they should be at least 90%, preferably higher. Although all agreed that ideally both sensitivity and specificity would be high, some prioritised one over the other. Clinicians who prioritised sensitivity said they would want to ensure all cases were detected and would not want to be dissuaded from providing treatment. However, others emphasised the need for a highly specific test to reduce false positive diagnoses and potentially missing another cause of symptoms.

Clinicians considered when they would use a diagnostic test. Some reported often feeling uncertain about a PID diagnosis and discussed how a test may be useful to enhance and support their clinical diagnosis. However, others highlighted that a test would not be useful for patients with obvious signs of PID. Importantly, many clinicians emphasised that no test would replace their clinical judgement. It was generally felt it would be better to dismiss a negative test result if the patient had met the clinical diagnostic criteria for PID, especially if treatment had been initiated and symptoms were improving. For most clinicians, a diagnostic test was regarded as being particularly useful for patients with less clear signs and symptoms. When managing some patients with low level symptoms, even highly experienced clinicians reported feelings of doubt about a PID diagnosis, and a diagnostic test was seen to be most useful for this group of patients.

#### Impact of a test on time to treatment

Clinicians discussed how diagnosing and providing treatment for patients in a timely manner is important, especially for patients unlikely to return for review. Most said they would treat empirically when suspicious of PID, although others indicated that they might offer an ultrasound or wait for results from STI testing, especially if the patient’s pain was manageable. Clinicians considered how the availability of a diagnostic test for PID could impact the time to a patient receiving a diagnosis. All agreed that a test with a long turnaround time would not be particularly helpful. Clinicians were especially enthusiastic about a test being available as a point-of-care test. This was seen to be particularly important in settings where decisions needed to be made quickly (e.g. hospital EDs) or where patients might be lost to follow up (e.g. rural/remote areas). However, it was recognised by one clinician that there could be barriers to using a point-of-care diagnostic test in certain settings, especially general practice, where equipment requirements, maintenance, and costs may not be feasible. Another clinician also highlighted that if they needed to interpret results themselves from a point-of-care test then they may require some training, and that this would need to be a consideration when developing a test. As well as considering the impact of a test on time to diagnosis and treatment, some clinicians discussed how a test might also allow them to reach alternative diagnoses including chronic pain issues more quickly.

#### The patient experience is an important consideration for clinicians

Clinicians considered how a diagnostic test might affect patient experience. Some talked about specimen collection method, and an additional swab taken during a speculum examination was not perceived to be a barrier for symptomatic patients (who would be examined regardless) unless the collection method required a different, less comfortable type of swab. A self-collected vaginal swab for patients declining a pelvic examination was also seen to be potentially beneficial.

One clinician discussed how diagnostic tests, especially point-of-care tests, can provide a sense of reassurance and validation to symptomatic patients. However, others discussed how a test result would have the potential to contradict and undermine their clinical diagnosis, which could be confusing for the patient. In situations where a test result would be dismissed in favour of clinical judgement, clinicians expressed concerns that patients may question the justification of being offered a test.

All clinicians also considered cost to the patient to be highly important, especially considering women at risk of PID are often young and financially unstable. The perceived acceptable level of cost varied. Some clinicians working in sexual health clinics said patients would not pay or expect to pay for a test, while GPs generally thought a test costing around $20-$50 would be acceptable. However, one clinician did consider how a test might reduce overall cost for some, if it meant they did not need to access additional costly tests such as an ultrasound.

### Interviews with young women

Two themes were identified as discussed below with illustrative quotes provided in Table 3.

**Table 3:** Themes and illustrative quotes from interviews with young women.

Theme	Illustrative quotes
**Acceptance of a test for PID if presenting with symptoms**	*I’d definitely want to know more about what* [PID] *is*, *just so I know what could happen*. *I’d rather go into* [a pelvic examination / test] *knowing what I could have*, *yeah*, *definitely*. #9 (24 years)
	*That’s something that would be*, *at least for me*, *I would really want to know about*, *you know the process of*, *if it’s negative*, *if it’s positive and you know*, *what to do with the antibiotics and stuff like that*. #5 (25 years)
	*At the moment*, *I’m not really earning enough to pay for a lot of my medical expenses*, *so I have to wait until I can find Medicare bulk billed options*. *So*, *if I had to pay out of pocket for that kind of test*, *I probably wouldn’t be able to pay for it and I would have to have an alternative test*, *so I’d probably have to rely on the clinician diagnosis*. #8 (27 years)
**The role of the clinician**	*I would say a really reassuring female nurse or doctor*. *That would play a huge role*, *because obviously if it was a male*, [I] *definitely wouldn’t get it* [a pelvic examination] *done*. #3 (21 years)
	*As long as they treated me with respect and explained the situation*, *I would feel comfortable regardless of gender or how many times I’ve previously seen them*. #8 (27 years)
	*I mean if I went in with just the thought that I might have an STI*, *that* [a speculum examination with a clinician-collected PID test] *might*, *like*, *I guess not be the most convenient thing*. *But if it was recommended by my doctor and they thought I should check anyway*, *I would definitely just do it*. #1 (21 years)
	*I would pay*, *like*, *$500* [if doctor said it would be a good idea to have a PID test]. *Like*, *that to me is like*, *“oh*, *you need to get it done*.*”* #3 (21 years)
	*If you have a clinician who knows what they’re doing or who actually cares about their patients*, *they actually look into things*, *and they will follow it through to actually provide quality care for their patients*. *They’re the ones that you can trust to actually discuss things properly and appropriately with you to find a solution*, *and they’re the ones that I would want to*, *sort of*, *consult with to find the best option*. #8 (27 years)

Quotes accompanied by participant number and age

#### Acceptance of a test for PID if presenting with symptoms

Young women were asked about their acceptance of a pelvic examination and new test for PID if they were experiencing symptoms suggestive of this diagnosis (new onset pelvic pain and abnormal vaginal discharge were symptoms presented in hypothetical scenarios). Not all women said that they would be expecting a pelvic examination, even when presenting to their GP with symptoms, and there were some preconceptions about what an examination would involve. Most thought they would agree to a pelvic examination, although almost all stipulated that this would need to be conducted by a female clinician. However, some were apprehensive about the prospect of an examination that involved a speculum, with the perception that it would be painful. Most women said they would not expect a bimanual pelvic examination to be included, and some considered this to be particularly intimate or uncomfortable. Two women said they would probably not agree to a pelvic examination within the initial consultation, although one said that if she was prepared, she might return for an examination later. The other woman said that she would want to seek a second opinion to determine if an examination was absolutely necessary.

Those who said they would agree to an examination said the inclusion of a PID test, perhaps involving an additional swab during the examination would be acceptable. Although most preferred the idea of a clinician-collected swab rather than self-collected, to minimise concerns about user-error, a few women did say they would like the option of a self-collected swab, providing this was accurate and easy for them to perform.

Although tests and investigations suggested to young women were generally considered acceptable, it was also clear that women would need to feel that an examination and test was justified, and many discussed that they would need to receive clear information about PID and a test. While some women said they would not want extensive information until after a diagnosis to prevent unnecessary worrying, all felt that at least some key information would be needed to assure them that a test was warranted. Some highlighted specific information they would want to know, including the causes of PID, potential complications, and treatment (including side effects). A few women said there were specific things they would want to know about a test, including accuracy/performance and details of how it would work.

All women said that cost would be important when considering whether they might accept a test. Although most women said they would be prepared to pay for a test, the amount that they would be willing to pay varied greatly. When asked about how they would feel if a PID test cost an additional fee, some struggled to see the benefit of paying extra, as there is an alternative way to diagnose PID that would be cheaper or free. Those who said they would pay an additional fee were asked the maximum amount that they would be likely to find acceptable. This ranged from $30 (AUD) to hundreds of dollars. Turnaround time to receiving results was not raised by any young women as something that might affect their decision-making when being offered a PID test. However, when asked about how long they would be prepared to wait for test results, most said they expect to receive a result within a week.

#### The role of the clinician

Women indicated they would need to trust and have rapport with their clinician to feel comfortable with any test requiring a pelvic examination, and many women preferred the idea of a female clinician. While sometimes trust was related to having an existing clinician-patient relationship, this was not perceived to be necessary for all women. More important was a clinician who presents as informative and non-judgemental, and who can create a relaxed environment.

A clinician’s judgement and recommendation were considered to be important factors that would influence decision-making. Most women suggested that if their clinician strongly advocated for a test then they would agree to have one, even if they felt it to be inconvenient, expensive, or unexpected. Women also discussed making decisions about commencing treatment before receiving a test result, and while many held reservations about doing so (most were concerned about side effects), a competent and trustworthy clinician’s strong support was seen to be persuasive.

## Discussion

Our findings indicate there is enthusiasm among clinicians for a highly accurate, minimally invasive, affordable test to diagnose PID. During interviews with clinicians, barriers to using a test were recognised and discussed, including the potential for results to contradict their clinical assessment, and the potential impact on time to treatment. All clinicians acknowledged that there is a degree of uncertainty when diagnosing PID, and some considered how diagnosis can be especially challenging for clinicians with less experience. This perception is reflected in other literature; some Australian clinicians who infrequently perform bimanual pelvic examinations have reported low levels of confidence in interpreting findings, [9] and less experienced clinicians have demonstrated lower levels of diagnostic ability for PID [8].

Clinicians interviewed in our study considered settings where a test for PID may be particularly beneficial, including rural and remote areas where PID has been found to be under-diagnosed in Australia [16]. Another setting suggested by multiple clinicians was hospital EDs. One clinician provided anecdotal evidence that PID presentations to their local hospital’s ED have been misdiagnosed as appendicitis. This clinician said that ED clinicians may not consider PID in women with lower abdominal pain, and face barriers to talking with patients about sexual health. There is little existing Australian research that explores ED clinicians’ PID diagnostic skills, although ED paediatricians demonstrated low levels of knowledge of PID diagnosis and treatment in a US hospital [17]. Considering that women with recent onset pelvic pain may present to hospital EDs, and the ED is where a significant amount of PID is diagnosed in Australia, [2] this is a setting that should be considered when developing and evaluating a diagnostic test for PID.

While clinicians discussed where a diagnostic test would be useful, they also considered to what extent they would allow results to impact patient management. Clinicians said results would be carefully considered and contribute to their decision-making, particularly when managing patients with less clear-cut symptoms. Importantly though, most emphasised no test would replace clinical judgement, indicating that a clinical assessment and diagnosis would remain important when managing patients. When considering how to use, interpret, and apply results of diagnostic tests, clinicians should consider several factors including the pre-test probability of disease, accuracy of a test, and potential harms of the test [18]. Clinicians interviewed were mindful of test performance factors, and clearly expressed how diagnostic tests can be used as a tool to support diagnosis, but should not be relied on entirely.

Our findings also suggest that young women would be accepting of a test, although women interviewed had limited knowledge of PID and would rely heavily on their clinician’s advice. Although for some of our interviewees, an existing relationship was considered preferable when receiving intimate examinations, a finding that has been reported elsewhere, [19] others discussed how trust is not necessarily dependent on an existing relationship, but can be quickly established within a consultation. While women had differing opinions about the amount of information they would want to receive about PID and a test for PID, it was clear they would need enough information to feel that the test was justified. The justification of paying an additional cost for a test was also a primary concern. Young women highlighted the importance of cost, with some saying that they would assume that such a test would be free. Some questioned whether they would pay an additional cost for a PID test if their clinician could investigate and diagnose them clinically. Clinicians were highly aware that cost would impact a patient’s decision to accept a test, and some noted that women at higher risk of STIs and PID are younger, and often less financially stable, a perception supported by research that has found that age and socioeconomic status impact on the risk of STI and PID acquisition [20–22]. Therefore, in order to be accessible and justifiable to patients who would benefit most, a diagnostic test for PID would need to be as cheap as possible, preferably free.

Our study had several key strengths and limitations. Firstly, this study is the first in Australia to explore the acceptability of a diagnostic test for PID and provides important insight into some of the key characteristics that end users would want a test to have in order for it to be useful in the primary care setting. It is strengthened by including both clinician and consumer end-user views. The primary limitation of our study is that we utilised a convenience sample, and all participants were self-selected. Our findings suggest that clinicians with less experience or confidence with diagnosing PID may find a test particularly beneficial, including those in non-specialised settings such as general practice and busy emergency departments. However, due to our sampling method and selection criteria, we did not interview any clinicians with little or no experience with diagnosing PID, and many of our interviewees were relatively confident with their diagnostic ability. This is unsurprising, as clinicians who responded to the study advertisement are likely to have been interested in sexual and reproductive health. We interviewed only one male clinician and it is clear that male GPs face additional barriers to conducting pelvic examinations; our previous research found male GPs less likely to perform pelvic examinations on symptomatic women than female GPs [9]. Our findings are therefore not generalisable to all Australian primary care clinicians, and we were not able to explore the perspectives of clinicians working in a wide range of clinical settings.

Young women who responded to our advertisement were a largely homogenous group; all higher education students living in metropolitan areas in the same state. As this group of interviewees included only young students, it should be recognised that perspectives on preparedness to pay for a test may not be generalisable to older, employed women with more capacity to pay. As with our clinician respondents, utilising a convenience sample is a key limitation of this study. The women who chose to respond to our advertisement may have been more open about talking about sexual health, and it is possible they had a particular interest in sexual and reproductive health and/or PID. This limits the generalisability of our findings to other young Australian women.

We recruited sexually active young women rather than patients or individuals with a history of PID as this study was exploring how this population (who are at the highest risk of PID) might feel when offered a diagnostic test for PID by a clinician when presenting with symptoms of an STI. We did not ask young women participants any questions about their medical or sexual history. While some respondents volunteered details during the interviews, and appeared to consider the hypothetical scenarios presented to them in the context of their own experiences, not all respondents did so. We therefore cannot determine to what extent participants relied on their own experiences when discussing their perspectives on being offered a test for PID. In addition to this, while efforts were made to provide the young women participants with clear information about PID, most had not previously heard of PID, and some young women may have had limited capacity to engage with the hypothetical scenarios presented to them. Although it was felt that clinician participants engaged well with the scenarios and questions posed to them, we also acknowledge that the clinicians were also discussing something hypothetical, and this is a further limitation of our study.

Finally, it should be acknowledged that some women experience ‘silent’ or sub-clinical PID [11]. Clinicians and women may have different views and concerns about the application of a diagnostic test for asymptomatic patients. However, the use of a test for asymptomatic PID was beyond the scope of this paper.

## Conclusions

Our research into the perspectives of clinician and young women regarding the acceptability of a hypothetical diagnostic test for PID has highlighted that an objective test may be a useful tool to support clinicians when diagnosing PID in a symptomatic patient, particularly when managing a patient with mild symptoms where a definitive diagnosis is less clear. Women would be accepting of a test if a trusted clinician recommended it, although they would need to feel it is justifiable. Development of a diagnostic test that could be used in settings where PID is especially challenging to diagnose, including emergency departments, will be beneficial.

## Supporting information

S1 File Interview guides. (DOCX)
