# Supplementary material for: Is there a place for a molecular diagnostic test for pelvic inflammatory disease in primary care? An exploratory qualitative study
Source: PLoS One. 2022 Sep 19;17(9):e0274666. doi: 10.1371/journal.pone.0274666 (PMC9484633; doi:10.1371/journal.pone.0274666)
Supplement: S1 File — (DOCX) [file pone.0274666.s001.docx]

# **Overview of interview schedule for participating young women**

Online (Zoom) interviews will be conducted with young women, aged between 18-30 who are currently living in Australia. Interviews will be semi-structured, and the interview schedule will be adjusted as data collection progresses. Verbal consent will be gained prior to the commencement of the interview.

An overview of pelvic inflammatory disease and associated STIs (including causes, symptoms, diagnosis, and health consequences) will be provided throughout the interview where appropriate. Questions asked in the interview will broadly cover the following topics, and they will be asked about hypothetical situations (not their own experiences):

- **Demographics:** including age, gender identity, employment status, postcode.
- **Awareness of PID and STIs:** (After an explanation about PID and its association with some STIs) Had they heard of PID before, and were they aware of the health consequences that it has on young women?
- **Thoughts on STI testing methods:** including acceptability of cervical swabs and/or vaginal swab testing for STIs (as opposed to urine sample). If swab testing acceptable, the acceptability of including PID testing alongside STI testing. *Participants will be asked hypothetically about what is acceptable to them, and will not be asked to disclose whether they have been tested or diagnosed with any STIs.*
- **Acceptability of a speculum examination (with cervical swabs) vs. bimanual examination:** including thoughts on risk of delayed diagnosis vs. presumptive diagnosis, perspectives on pelvic examinations (speculum vs bimanual), and factors that might impact patient decision to consent to speculum and/or bimanual examinations (e.g. relationship with doctor, gender of doctor, menstruation, etc.)
- **Opinion on characteristics of test:** How do they feel about different types of tests (self-collected swab vs. collected by doctor), how important is the cost of the test (if they knew that the test might help to detect undiagnosed PID, would they be prepared to pay for it?), how important is the time to receiving test results, and would that impact on its level of acceptability to them?
- **What information about PID they would require:** (i) as a patient receiving STI testing; (ii) as a patient with symptoms. If they had a positive result, would they require more information about infertility (or possibly investigations into their fertility)?
- **Any additional information they would like to add**

# **Overview of interview schedule for participating clinicians**

Online (Zoom) interviews will be conducted with clinicians who are currently working in Australian general practice, sexual health clinics, or family planning clinics. Interviews will be semi-structured, and the interview schedule will be adjusted as data collection progresses. Verbal consent will be gained prior to the commencement of the interview.

Questions asked in the interview will broadly cover the following topics:

- **Demographics:** including age, gender identity, how long they have been working as a healthcare provider in Australia, how many hours they work per week, postcode of current workplace, did they train in Australia? If not, where? If they trained overseas how long have they been working in Australia? Any additional training or education in sexual and reproductive health?
- **Experience diagnosing and managing STIs and PID:** including how often they test and diagnose female patients for STIs (specifically chlamydia and gonorrhoea), and how many cases of PID they diagnose in an average year. How do they normally test for chlamydia and/or gonorrhoea (cervical swabs / patient-collected vaginal swabs / urine sample)? Do they normally perform pelvic examinations on patients with symptoms of pelvic inflammatory disease (bimanual, speculum, or both)? Have their practices around diagnosing and managing STIs and PID changed due to COVID-19?
- **Opinion on usefulness of a diagnostic test for PID for symptomatic patients:** Will a diagnostic test be useful to them in their practice when they are treating a patient with symptoms of PID (why/why not?). Would they still be likely to perform a bimanual pelvic examination if the test was available, or would they use a diagnostic test instead of performing a bimanual examination?
- **Opinion on usefulness of a diagnostic test for PID for asymptomatic patients who are receiving STI testing:** Would they consider testing patients for PID at the same time as other STIs (why/why not?). Would their feelings about this change depending on the type of test available (e.g. one swab for multiple STIs, self-collected vaginal swab)? Would they be willing to accept the result of a positive test for PID and prescribe treatment for patients with minimal symptoms *without* conducting further investigations?
- **Other factors that would make a test acceptable/useable to them, and their patient:** including the test turnaround time (TAT) and how that would impact on their decision to use the test, the optimal performance and reliability of the assay (sensitivity and specificity of the test), and whether they would be likely to use the test if it incurred an additional cost to their patient?
- **Any additional information they would like to add**
